# Detecting discordance enrichment among a series of two-sample genome-wide expression data sets

**DOI:** 10.1186/s12864-016-3265-2

**Published:** 2017-01-25

**Authors:** Yinglei Lai, Fanni Zhang, Tapan K. Nayak, Reza Modarres, Norman H. Lee, Timothy A. McCaffrey

**Affiliations:** 1 Department of Statistics, The George Washington University, 801 22nd St. N.W., Rome Hall, 7th Floor, Washington, 20052 D.C. USA; 2 Department of Pharmacology and Physiology, The George Washington University Medical Center, Washington, 20037 D.C. USA; 3 Department of Medicine, Division of Genomic Medicine, The George Washington University Medical Center, Washington, 20037 D.C. USA

**Keywords:** Discordance, Gene set enrichment, Mixture models

## Abstract

**Background:**

With the current microarray and RNA-seq technologies, two-sample genome-wide expression data have been widely collected in biological and medical studies. The related differential expression analysis and gene set enrichment analysis have been frequently conducted. Integrative analysis can be conducted when multiple data sets are available. In practice, discordant molecular behaviors among a series of data sets can be of biological and clinical interest.

**Methods:**

In this study, a statistical method is proposed for detecting discordance gene set enrichment. Our method is based on a two-level multivariate normal mixture model. It is statistically efficient with linearly increased parameter space when the number of data sets is increased. The model-based probability of discordance enrichment can be calculated for gene set detection.

**Results:**

We apply our method to a microarray expression data set collected from forty-five matched tumor/non-tumor pairs of tissues for studying pancreatic cancer. We divided the data set into a series of non-overlapping subsets according to the tumor/non-tumor paired expression ratio of gene *PNLIP* (pancreatic lipase, recently shown it association with pancreatic cancer). The log-ratio ranges from a negative value (e.g. more expressed in non-tumor tissue) to a positive value (e.g. more expressed in tumor tissue). Our purpose is to understand whether any gene sets are enriched in discordant behaviors among these subsets (when the log-ratio is increased from negative to positive). We focus on KEGG pathways. The detected pathways will be useful for our further understanding of the role of gene *PNLIP* in pancreatic cancer research. Among the top list of detected pathways, the neuroactive ligand receptor interaction and olfactory transduction pathways are the most significant two. Then, we consider gene *TP53* that is well-known for its role as tumor suppressor in cancer research. The log-ratio also ranges from a negative value (e.g. more expressed in non-tumor tissue) to a positive value (e.g. more expressed in tumor tissue). We divided the microarray data set again according to the expression ratio of gene *TP53*. After the discordance enrichment analysis, we observed overall similar results and the above two pathways are still the most significant detections. More interestingly, only these two pathways have been identified for their association with pancreatic cancer in a pathway analysis of genome-wide association study (GWAS) data.

**Conclusions:**

This study illustrates that some disease-related pathways can be enriched in discordant molecular behaviors when an important disease-related gene changes its expression. Our proposed statistical method is useful in the detection of these pathways. Furthermore, our method can also be applied to genome-wide expression data collected by the recent RNA-seq technology.

## Background

Genome-wide expression data have been widely collected by the recent microarray [1–3] or RNA-seq technologies [4, 5]. In addition to the differential expression analysis for the identification of potential study-related biomarkers [6], gene set enrichment analysis (or gene set analysis) for the identification of study-related pathways (or gene sets) has received a considerable attention in the recent literature [7, 8]. It enables us to detect weak but coherent changes in individual genes through aggregating information from a specific group of genes.

In the current public databases, large genome-wide expression data sets or multiple genome-wide expression data sets have been made available [3, 9]. For a large data set, multiple subsets can be generated according to different stages of an important feature. Integrative analysis enables us to detect weak but coherent changes in individual datasets through aggregating information from different datasets [10–12].

Integrative gene set enrichment is an approach that aggregates information from a specific group of genes among different datasets [13–15]. Due to the aforementioned complex analysis scenario, different analysis methods are needed to address different study purposes. For example, the study purpose can be to identify gene sets with statistical significance after data integration (without considering whether changes are positive or negative) and an extension of traditional meta-analysis method can be used, or the study purpose can be to identify gene sets with concordance enrichment and a mixture model based approach can be used.

In this study, we consider a series of related genome-wide expression data sets collected at different stages of an important feature. For an illustrative example, RNA-seq data can be collected at many different growth time points and we are interested in the following study purpose. The gene expression in some pathways may be overall high at early time points and overall low at later time points. It is biologically interesting to identify these pathways with clearly discordant behaviors. Pang and Zhao [16] have recently suggested a stratified gene set enrichment analysis. (Jones et al. [17] also recently conducted a stratified gene expression analysis.) The analysis purpose in this study is different from theirs. As we have explained, to achieve an efficient analysis for the detection of discordance among a series of related genome-wide expression data sets, we need a specific statistical method.

In a differential expression analysis and/or gene set enrichment analysis, it is usually unknown whether a gene is truly differentially expressed (up-regulated or down-regulated) or non-differentially expressed (null). Statistically, we can conduct a test (e.g. *t*-test) for the observations from each gene and obtain a *p*-value to evaluate how likely the gene is differentially expressed. False discovery rate [6, 18] can be used to evaluate the proportion of false positives among claimed positives. Another approach can also be considered. It is based on the well-known finite normal-distribution mixture model [19]. Signed *z*-scores can be obtained from one-sided *p*-values [15, 20]. The assumption is that all the *z*-scores are a sample of a mixture model with three components: one with zero population mean representing non-differentially expressed genes and the other two with positive and negative population means representing up-regulated and down-regulated genes, respectively. The false discovery rate (FDR) can be conveniently calculated under this framework.

In the mixture model approach, although the component information is still unknown, it can be estimated by the well-established E-M algorithm [19]. This information has been used to address the enrichment in concordance among different data sets [15]. In this study, our interest is to detect enrichment in discordance among a series of related genome-wide expression data sets collected at different stages of an important feature. The estimated component information can be useful in the calculation of discordance enrichment probability (see “Methods” for details). Therefore, our method is developed based on a mixture model.

In the “Methods” section, we will review the background for our mixture model based approach. Without a structure consideration, the model parameter space increases exponentially with the increase of number of data sets. Therefore, a novel statistical contribution of this study is that we propose a two-level mixture model to achieve a linearly increased parameter space with the increase of number data sets. The model parameters can be estimated by the well-established E-M algorithm and the model-based probability of discordance enrichment can be calculated for gene set detection.

## Motivation

Table 1 gives an artificial example to illustrate discordance enrichment. Assume there are six two-sample genome-wide expression data sets, and *z*-scores (see “Methods” for details) for all genes are calculated. Assume there is an important molecular pathway with nine genes, and their *z*-scores are shown in Table 1. A positive or negative *z*-score implies a possible up-regulation or down-regulation, respectively. In Table 1, there are several genes with some clearly positive and some clearly negative *z*-scores (like absolute value greater than 4). For examples, *z*-scores 7.7, 4.8, -4.9 and -7.6 are observed for gene *G*_4_; *z*-scores 6.5 and -8.1 are observed for gene *G*_5_; *z*-scores 7.9, 5.0, 4, -8.6 and -8.9 are observed for gene *G*_6_; *z*-scores 4.6, -5.6 and -9.0 are observed for gene *G*_7_, and *z*-scores 5.3, -4.1 and -4.8 are observed for *G*_8_. These observations of clear discordance suggest that, in this pathway, some genes may behave clearly differently among different data sets. Furthermore, there are five out of nine genes with these clear discordant behaviors. If we only expect about 30*%* of genes with such behaviors, then this proportion is obviously large (>50*%*). An exploration of pathways (or gene sets) enriched in clear discordance will enable us to further understand the molecular mechanisms of complex diseases.

**Table 1:** An artificial example for discordance illustration

	*z*-Score					
Gene	*z*_1_	*z*_2_	*z*_3_	*z*_4_	*z*_5_	*z*_6_
*G*_1_	6.4	8.8	6.8	8.4	10.4	1.2
*G*_2_	5.2	4.5	7.0	5.5	3.3	6.3
*G*_3_	-0.3	-1.9	1.8	2.9	6.7	1.5
*G*_4_	4.8	7.7	2.3	-4.9	-7.6	2.2
*G*_5_	1.9	6.5	-1.2	0.9	-8.1	2.1
*G*_6_	4.0	-8.9	-1.1	5.0	-8.6	7.9
*G*_7_	3.7	-5.6	-1.6	-0.6	-9.0	4.6
*G*_8_	-3.1	-4.8	-1.6	5.3	-2.9	-4.1
*G*_9_	-6.3	-9.7	-1.1	-6.4	-8.4	-7.1

Pancreatic cancer related studies are important in public health [21]. Recently, gene *PNLIP* (pancreatic lipase) has been shown its association with the pancreatic cancer survival rate [22]. A paired two-sample microarray genome-wide expression data set has been collected for studying pancreatic cancer [23]. One advantage of this paired design is that we can focus on the expression ratio between tumor and non-tumor tissues for each gene. One related biological motivation is to use the genome-wide expression data set to understand molecular changes related to the change of expression ratio of gene *PNLIP*. In this study, more specifically, our interest is to identify pathways or gene sets showing clearly discordant behavior when the expression ratio of gene *PNLIP* changes. Understanding these molecular changes can help us further investigate the role of gene *PNLIP* and even the general disease mechanism of pancreatic cancer.

Gene expression profiles are measured as continuous variables. However, if we can perform this analysis with a relatively simple method, then the results can be more interpretable. Therefore, our approach is to divide the microarray data set into a series of non-overlapping subsets according to the tumor/non-tumor paired expression ratio of gene *PNLIP*. The log-ratio ranges from a negative value (e.g. more expressed in non-tumor tissue) to a positive value (e.g. more expressed in tumor tissue). Our purpose is to understand whether any gene sets are enriched in discordant behaviors among these subsets (when the log-ratio is increased from negative to positive). Notice that we only use the expression ratio of gene *PNLIP* to divide the study data set. We do not consider the expression profiles of other genes for data division. There is no analysis optimization in data division and this strategy avoids the selection bias towards our analysis.

The number of study subjects in the microarray data set is adequate so that we can divide the data set into many subsets (e.g. greater than five) so that the biological changes can be better explored. After dividing the study data set into *K* non-overlapping subsets, we can perform genome-wide differential expression analysis for each subset. Genes can be generally categorized as up-regulated (positively differentially expressed), down-regulated (negatively differentially expressed) or null (non-differentially expressed). Genes may show concordant behaviors or discordant behaviors among different subsets. For examples, showing positive differential expression in all *K* subsets is clearly a concordant behavior and showing negative differential expression in the first subset but positive differential expression in the last subset is clearly a discordant behavior.

In a genome-wide differential expression analysis, we usually calculate the test scores based on a chosen statistic (e.g. *t*-test) to evaluate whether genes are differentially expressed or not. For simplicity, we choose the well-known two-sample *t*-test. A strong positive or negative differential expression would result in clearly positive or negative test score. A non-differential expression would result in a test score close to zero and the test score could be either positive or negative (but rarely zero exactly). Therefore, if a gene is concordantly differentially expressed (e.g. all up-regulated with clearly positive test scores) in some subsets but it is not differentially expressed (e.g. all null with slightly positive test scores) in the other subsets, then it can be statistically difficult to evaluate whether the gene has an overall discordant behavior.

Therefore, in this study, we focus on genes with some clearly discordant behaviors: up-regulated in at least one subset and down-regulated in at least one subset (to avoid the statistical difficulty mentioned above). We are interested in identifying pathways or gene sets enriched in clearly discordant behaviors. We focus on KEGG pathways. The detected pathways will be useful for our further understanding of the role of gene *PNLIP* in pancreatic cancer research.

Gene *TP53* is well-known for its role as tumor suppressor in general cancer studies. Its log-ratio in the microarray data set also ranges from a negative value (e.g. more expressed in non-tumor tissue) to a positive value (e.g. more expressed in tumor tissue). We also divide the microarray data set according to the expression ratio of gene *TP53* and repeat the discordance enrichment analysis. We consider the analysis result based on gene *TP53* a useful comparison with the analysis result based on gene *PNLIP*.

## Methods

### Multiple data sets

In this study, we consider a detection of gene set enrichment in discordant behaviors (or discordance gene set enrichment) for a series of two-sample genome-wide expression data sets. The term “enrichment in discordant behaviors" will be mathematically defined later. Let *K* be the number of data sets and let *m* be the number of common genes among this series of data sets. Each data set is collected for two given groups (same for all *K* data sets). In general, one group represents a normal status and the other group represents an abnormal status.

For a single two-sample genome-wide expression data set, differential expression analysis and gene set enrichment analysis are usually conducted. The purpose of analysis of differential expression is to identify genes showing significantly up-regulation or down-regulation when two sample groups are compared. The purpose of gene set enrichment analysis is to identify pathways (or gene sets) showing coordinate up-regulation or down-regulation, which may be considered as an extension of differential expression analysis.

Therefore, the following gene behaviors are usually of our research interest in two-sample expression data analysis: positive change (or up-regulation), negative change (or down-regulation) and null (or non-differentially expressed). However, these underlying behaviors are usually not observed and expression data are collected to make statistical inference about them.

Data pre-processing is important for both microarray and RNA-seq data and it has been well discussed in the literature [24–26]. In our study, the data can be downloaded from a well-known public database. We assume that the gene expression profiles have been appropriately pre-processed. In an analysis of multiple expression data sets, it is usually necessary to focus on common genes and gene identifiers can be useful for this purpose. In our study, we divide a relatively large data set into a series of non-overlapping subsets. Therefore, all the genes in the downloaded data are common.

### ***z***-Score

Many statistical tests have been proposed for analyzing a two-sample genome-wide expression data set [27, 28]. In this study, the traditional paired-two-sample *t*-test is chosen for its simplicity (although other statistics could be certainly considered, see below). For each gene in each data set (or subset), we perform the *t*-test to obtain a *t*-score. Its *p*-value is evaluated based on the permutation procedure (randomly switch the tumor/non-tumor labels for each pair of tissues) so that the normal distribution assumption is not assumed for the paired-difference data. All the permuted *t*-scores are pooled together so that tiny *p*-values can be calculated [29].

One-sided upper-tailed *p*-values are calculated so that the direction of change can be distinguished for each gene in each data set. Let *p*_*i,k*_ be the *p*-value for the *i*-th gene in the *k*-th data set. *z*-scores are obtained by an inverse normal transformation

$$z_{i,k} = \Phi^{-1}(p_{i,k}), $$

where *Φ*(·) is the cumulative distribution function (c.d.f.) of the standard normal distribution (mean zero and variance one). This transformation has been widely used [20] and our proposed multivariate normal mixture model will be applied to the transformed *z*-scores.

### Discordance enrichment

Our proposed method is a type of gene set enrichment analysis. As it has been discussed by Lai et al. [15], we defined “enrichment” as “the number of events of interest is larger than expected” and our “event of interest” in this study is “a list of clearly discordant behaviors” from a gene. If we know whether the expression profile of a gene is up-regulated (simplified as “up”), down-regulated (simplified as “down”) or non-differentially expressed (simplified as “null”) in a data set, then a list of concordant behaviors among *K* data sets for this gene could be (up, up, …, up), (down, down, …, down) or (null, null, …, null). In this study, we focused on a list with at least one “up” and at least one “down” among *K* data sets. For example, a list like (down, up, up, …, up) is an event of interest but a list like (null, up, up, …, up) is not. The reason is “down” and “up” can be visually distinguished by the negative (“-") and positive (“+”) signs in *z*-scores, respectively. However, zero *z*-scores are rarely observed. Therefore, it is less clear to distinguish “null” from “up” (or “null” from “down”).

Based on the expression profiles, we obtain *z*-scores to make statistical inference about genes’ behaviors in each data set. To evaluate “discordance enrichment" as defined above, we considered a mixture model approach that allows us to estimate the probability of a behavior (“up”, “down” or “null”) and the expected number of events of interest (notice that these are not directly observed in the data sets). Let *S* be the set of genes for a pathway (or gene set in general) and *m*_*S*_ the number of genes in *S*. If the *i*-th gene in *S* is showing a list of clearly discordant behaviors, then we set an indicator variable *U*_*S,i*_=1; Otherwise, we set *U*_*S,i*_=0. Then, we can calculate the discordance enrichment score (DES) for gene set *S* that is a probability defined as

$$DES_{S} = \mathbf{Pr}\left(\sum_{i=1}^{m_{S}} U_{S,i} > m_{S} \theta\right), $$

in which *θ* is the proportion of genes with clearly discordant behaviors.

In our mixture model, we used normal distributions to model the *z*-scores. A novel contribution is that the parameter space of our model increases linearly when the number of data sets is increased. This is due to the two-level structure of our model. (The parameter space of a general model for this analysis increases exponentially when the number of data sets is increased). For each gene in each data set, we considered three normal distribution components that represent up-regulation (positive distribution mean), down-regulation (negative distribution mean) and null (zero mean). (Theoretically, *p*-values under the null hypothesis are uniformly distributed. Therefore, *z*-scores under the null hypothesis are normally distributed with mean zero and variance one). The mathematical details are described below.

### A two-level mixture model

First, we described the basic model structure for just one data set. Then, we introduced our novel two-level mixture model. A simple three-component normal distribution mixture model [30, 31] is considered for each *z*-score *z*_*i,k*_ (the *i*-th gene in the *k*-th data set, *i*=1,2,…,*m* and *k*=1,2,…,*K*):

$$f(z_{i,k}) = \sum_{j_{k}=0}^{2} \rho_{j_{k},k} \phi_{\mu_{j_{k},k}, \sigma^{2}_{j_{k},k}}(z_{i,k}). $$

In the above model, $\phantom {\dot {i}\!}\phi _{\mu, \sigma ^{2}}(\cdot)$ is the probability density function (p.d.f.) of a normal distribution with mean *μ* and variance *σ* ^2^. Three components represent up-regulation with *μ*_1,*k*_>0, down-regulation with *μ*_2,*k*_<0 and null with *μ*_0,*k*_=0 (also recall that $\sigma ^{2}_{0,k}=1$). For this model, an assumption is that the p.d.f. of *z*_*i,k*_ is simply $\phi _{\mu _{j_{k},k}, \sigma ^{2}_{j_{k},k}}(z_{i,k})$ if we know the underlying component information *j*_*k*_ for the *i*-th gene in the *k*-th data set. However, the component information is usually not observed in practice. Then, we have this one-dimensional mixture model after the introduction of component proportion parameters $\left \{ \rho _{j_{k},k}, j_{k}=0,1,2 \right \}$ for the *k*-th data set.

When we extend the above mixture model to a higher dimension (i.e. *K* data sets), without a structure consideration, the parameter space increases exponentially due to the 3^*K*^ different component combinations (3 components in each of *K* data sets). Therefore, when *K* is not a small number (i.e. *K*>4), we need a more efficient model [15]. Biologically, when different data sets are collected for the same or similar research purpose, some genes are likely to show consistent behaviors across different data sets and some genes are likely to show different behaviors. For genes likely showing consistent behaviors across *K* data sets, we consider a complete concordance (CC) multivariate model to approximate the distribution of {*z*_*i,k*_,*k*=1,2,…,*K*}. For genes likely showing different behaviors across *K* data sets, we consider a complete independence (CI) multivariate model to approximate the distribution of {*z*_*i,k*_,*k*=1,2,…,*K*}. (Notice that there is no overlap among multiple data sets. If the component information among these data sets is known, then *z*-scores are independent.) We first describe the CI model and CC model as below.

The CI model assumes that the behaviors of the *i*-th gene are independent across different data sets. Therefore, we have the following mixture model:

$$f_{CI}(z_{i,1}, z_{i,2}, \ldots, z_{i,K}) = \prod_{k=1}^{K}\left[\sum_{j_{k}=0}^{2} \rho_{j_{k},k} \phi_{\mu_{j_{k},k},\sigma^{2}_{j_{k},k}}(z_{i,k})\right]. $$

This model is simply a product of *K* one-dimensional three-component mixture-models.

The CC model assumes that the behaviors of the *i*-th gene are the same across different data sets. Although the component information is unknown, the components for different data sets must be consistent. Therefore, we have the following mixture model:

$$f_{CC}(z_{i,1}, z_{i,2}, \ldots, z_{i,K})=\sum_{j=0}^{2}\left[\pi_{j} \prod_{k=1}^{K}\phi_{\mu_{j,k},\sigma_{j,k}^{2}}(z_{i,k})\right]. $$

This model has three components and each component is a product of *K* normal probability density functions.

In practice, it is unknown whether the *i*-th gene is showing independent or consistent behaviors. Therefore, we consider CI and CC as two high-level components and propose the following two-level model for {*z*_*i,k*_,*k*=1,2,…,*K*}:

$$\begin{aligned} f(z_{i,1}, z_{i,2}, \ldots, z_{i,K}) &= \lambda f_{CC}(z_{i,1}, z_{i,2}, \ldots, z_{i,K}) \\ & \quad+ (1-\lambda) f_{CI}(z_{i,1}, z_{i,2}, \ldots, z_{i,K}). \end{aligned} $$

Notice that this two-level model is still a mixture model. We further assume that $\{ \mu _{j_{k},k}, \sigma _{j_{k},k}^{2}, j_{k}=0,1,2, k=1,2,\ldots,K \}$ are shared by both CI and CC models. It is evident that the model parameter space increases linearly when the number of data sets (*K*) increases.

We can use the well-established Expectation-Maximization (E-M) algorithm [19] for parameter estimation. First, it is necessary to introduce some indicator variables (for component information) for the *z*-scores {*z*_*i,k*_,*k*=1,2,…,*K*} of the *i*-th gene. Then, we describe the E-step and M-step.

For high-level component information,

$${}\begin{aligned} \omega_{i}\,=\,\left\{\!\ \begin{array}{ll} 1 &\text{if gene's behaviors are consistent with CC model;}\\ 0 &\text{if gene's behaviors are consistent with CI model.} \end{array} \right. \end{aligned} $$

For CI model component information,

$${}\begin{aligned} \eta_{i,j_{k},k}=\left\{ \begin{array}{ll} 1 &\text{if \(z_{i,k}\) is sampled from the \(j_{k}\)-th component;}\\ 0 &\text{otherwise.} \end{array} \right. \end{aligned} $$

For CC model component information,

$${}{\begin{aligned} \xi_{i,j}=\left\{ \begin{array}{ll} 1 &\text{if all}\, \{ z_{i,k}, k=1,2,\ldots,K \}\, \text{are sampled from the}\, j\text{-th component;}\\ 0 &\text{otherwise.} \end{array} \right. \end{aligned}} $$

The E-step is the calculation of the following expected values when all the parameter values are given.

$${}{\begin{aligned} {\mathrm{E}}(\omega_{i})& = &\frac{\lambda f_{CC}(z_{i,1}, z_{i,2}, \ldots, z_{i,K})}{\lambda f_{CC}(z_{i,1}, z_{i,2}, \ldots, z_{i,K}) + (1-\lambda) f_{CI}(z_{i,1}, z_{i,2}, \ldots, z_{i,K})}, \end{aligned}} $$

$${}{\begin{aligned} {\mathrm{E}}((1&-\omega_{i}) \eta_{i,j_{k},k})\\ & = \frac{(1-\lambda)\rho_{j_{k},k}\phi_{\mu_{j_{k},k},\sigma_{j_{k},k}^{2}}(z_{i,k}) \prod_{h=1,h\neq k}^{K}\sum_{j_{h}=0}^{2}\rho_{j_{h},h}\phi_{\mu_{j_{h},h},\sigma_{j_{h},h}^{2}}(z_{i,h})}{\lambda f_{CC}(z_{i,1}, z_{i,2}, \ldots, z_{i,K}) + (1-\lambda) f_{CI}(z_{i,1}, z_{i,2}, \ldots, z_{i,K})}. \end{aligned}} $$

$${}\begin{aligned} {\mathrm{E}}&(\omega_{i} \xi_{i,j})\\ &= \frac{\lambda \pi_{j} \prod_{k=1}^{K} \phi_{\mu_{j,k},\sigma_{j,k}^{2}}(z_{i,k})}{\lambda f_{CC}(z_{i,1}, z_{i,2}, \ldots, z_{i,K})\! +\! (1-\lambda) f_{CI}(z_{i,1}, z_{i,2}, \ldots, z_{i,K})}, \end{aligned} $$

The M-step is the calculation of the following parameter values when all the component information is given:

$$\begin{array}{@{}rcl@{}} \hat{\lambda}&=&\frac{1}{m}\sum_{i=1}^{m}{\mathrm{E}}(\omega_{i}), \end{array} $$

$$\begin{array}{@{}rcl@{}} \hat{\rho}_{j_{k},k}&=&\frac{\sum_{i=1}^{m}{\mathrm{E}}\left((1-\omega_{i}) \eta_{i,j_{k},k}\right)}{\sum_{i=1}^{m}\sum_{j_{h}=0}^{2}{\mathrm{E}} \left((1-\omega_{i}) \eta_{i,j_{h},k}\right)}, \end{array} $$

$$\begin{array}{@{}rcl@{}} \hat{\pi}_{j}=\frac{\sum_{i=1}^{m}{\mathrm{E}}(\omega_{i} \xi_{i,j})}{\sum_{i=1}^{m}\sum_{h=0}^{2}{\mathrm{E}}(\omega_{i} \xi_{i,h})}, \end{array} $$

$$\begin{array}{@{}rcl@{}} \hat{\mu}_{j_{k},k}&=&\frac{\sum_{i=1}^{m}\left[{\mathrm{E}}(\omega_{i} \xi_{i,j_{k}})+{\mathrm{E}}((1-\omega_{i}) \eta_{i,j_{k},k})\right] z_{i,k}}{\sum_{i=1}^{m}[{\mathrm{E}}(\omega_{i} \xi_{i,j_{k}})+{\mathrm{E}}((1-\omega_{i}) \eta_{i,j_{k},k})]}, \end{array} $$

$$\begin{array}{@{}rcl@{}} \hat{\sigma}^{2}_{j_{k},k}\,=\,\frac{\sum_{i=1}^{m}[{\mathrm{E}}(\omega_{i} \xi_{i,j_{k}})+{\mathrm{E}}((1-\omega_{i}) \eta_{i,j_{k},k})]] (z_{i,k} - \hat{\mu}_{j_{k},k})^{2}}{\sum_{i=1}^{m}[{\mathrm{E}}(\omega_{i} \xi_{i,j_{k}})+{\mathrm{E}}((1-\omega_{i}) \eta_{i,j_{k},k})]}\!. \end{array} $$

E-step and M-step are iterated until a numerical convergence is achieved. In this study, the numerical convergence is defined as that the difference between the current log-likelihood and the previous one is within a given tolerance value (e.g. 10^−4^).

### Enrichment score

As we have discussed in *Discordance enrichment*, in this study, we focus on genes’ behaviors with at least one up-regulation and at least one down-regulation among *K* data sets (our event of interest: a gene with clearly discordant behaviors). However, we do not need to enumerate all these combinations (among 3^*K*^ in total). The related computing can be simplified if we enumerate the compliment events instead. There are three combinations for complete concordance: (up, up,..., up), (down, down,..., down) and (null, null,..., null). They will be excluded. There are $\sum _{l=1}^{K-1}{K \choose l}$ combinations with both “null" and “up" (without “down") and there are $\sum _{l=1}^{K-1}{K \choose l}$ combinations with both “null" and “down" (without “up"). They will also be excluded. Then, the remaining combinations are our events of interest (at least one “up" and at least one “down").

According to the above computing strategy, based on the two-level mixture model, the related proportion (*θ*) of genes with clearly discordant behaviors (also see “Discordance enrichment” for more details) can be calculated as follows.

$${}\begin{aligned} \theta\! =\! (1\,-\,\lambda)\left(\!1\! -\!\! \sum_{j=0}^{2} \prod_{k=1}^{K} \rho_{j,k} \,-\,\!\! \sum_{\{j_{k}\} \in A} \prod_{k=1}^{K} \rho_{j_{k},k} -\!\! \sum_{\{j_{k}\} \in B} \prod_{k=1}^{K} \rho_{j_{k},k}\! \right)\!, \end{aligned} $$

where *A* is the set of lists with a mix of 0’s and 2’s, and *B* is the set of lists with a mix of 0’s and 1’s.

Let *S* be a gene set with *m*_*S*_ genes. As defined in *Discordance enrichment*, let the indicator variable *U*_*S,i*_=1 if the *i*-th gene in *S* is showing a list of clearly discordant behaviors, and *U*_*S,i*_=0 otherwise. Then, based on the two-level mixture model, the related probability can be calculated as follows.

$${}\begin{aligned} \mathbf{Pr}(U_{S,i}=1) &= (1-\lambda)[ f_{CI}(z_{S,i,1}, z_{S,i,2}, \ldots, z_{S,i,K}) \\ & \quad- \sum_{j=0}^{2} \prod_{k=1}^{K} \rho_{j,k} \phi_{\mu_{j,k},\sigma^{2}_{j,k}}(z_{S,i,k}) \\ & \quad- \sum_{\{j_{k}\} \in A \cup B} \prod_{k=1}^{K} \rho_{j_{k},k} \phi_{\mu_{j_{k},k},\sigma^{2}_{j_{k},k}}(z_{S,i,k}) ] \\ & \quad / f(z_{S,i,1}, z_{S,i,2}, \ldots, z_{S,i,K}), \end{aligned} $$

where (*z*_*S,i*,1_,*z*_*S,i*,2_,…,*z*_*S*,*i,k*_) are the related *z*-scores. Let *ζ*_*S,i*_=**P** **r**(*U*_*S,i*_=1), which is a conditional probability according to the given model and observed data.

Under the assumption that *z*-scores from different genes are independent, the discordance enrichment score (DES) for gene set *S*, which has been defined in *Discordance enrichment* as $DES_{S} = \mathbf {Pr}\left (\sum _{i=1}^{m_{S}} U_{S,i} > m_{S} \theta ]\right)$, can be calculated as follows.

$$\begin{aligned} DES_{S} &= \sum_{U_{S,1}=0}^{1} \sum_{U_{S,2}=0}^{1} \cdots \sum_{U_{S,m_{S}}=0}^{1} \left[I\left(\sum_{i=1}^{m_{S}} U_{S,i}\right.\right.\\ & \left.\left. \quad > m_{S} \theta{\vphantom{\sum_{0}^{0}}}\right) \prod_{i=1}^{m_{S}} \zeta_{S,i}^{U_{S,i}} (1-\zeta_{S,i})^{1-U_{S,i}} \right], \end{aligned} $$

where *I*(true statement)=1 and *I*(false statement)=0 (indicator function). Since {*ζ*_*S,i*_,*i*=1,2,…,*m*_*S*_} are usually different for different genes, the above formula is a calculation of a tail probability for a heterogeneous Bernoulli process. The related computing issue and the related false discovery rate have already been discussed by Lai et al. [15]. Therefore, we described them briefly as below.

### False discovery rate

As discussed in the literature [15, 20], the above enrichment score is a conditional probability and a true positive proportion for gene set *S*. Therefore, the related false discovery rate [6, 18] for the top *T* gene sets {*S*_1_,*S*_2_,…,*S*_*T*_} identified by the above *DES* can be conveniently derived as below.

$$FDR = 1 - \sum_{t=1}^{T} DES_{S_{t}}/T. $$

### Computational approximation

As discussed in Lai et al. [15], the exact calculation of *DES* can be difficult due to the complexity of heterogeneous Bernoulli process. A Monte Carlo approximation has been suggested as follows. First, set an integer variable *X*=0. For the *i*-th gene in *S*, simulate a Bernoulli random variable with probability of event *ζ*_*S,i*_. Then, count the number of events from all genes in *S*, and increase *X* by one if this number is larger than *m*_*S*_ *θ*. Repeat the simulation and counting *B* times and report *X*/*B* as the approximated *DES*. *B*=2000 was suggested by Lai et al. [15].

## Results and discussion

### Genome-wide expression data and KEGG pathway collection

Zhang et al. [23] recently conducted a genome-wide expression study for forty-five matched pairs of pancreatic tumor and adjacent non-tumor tissues. The data were collected by the microarray technology (Affymetrix GeneChip Human Gene 1.0 ST arrays) and were made publicly available in the NCBI GEO database [23]. The collections of gene sets or pathways can be downloaded from the Molecular Signature Database [7, 8]. At the time of study, the collections have been updated to version 4.0. In this study, we focus on 186 KEGG pathways for our data analysis. There are 28677 genes available for our discordance enrichment analysis. As we have explained in the *Methods*, we expect to identify pathways with enrichment in clearly discordant gene behaviors among a series of pre-defined genome-wide expression data sets. (Notice that a pathway with *DES*∼1 is significantly enriched in clearly discordant behaviors; and a pathway with *DES*∼0 is evidently not enriched in clearly discordant behaviors).

### Data division based on gene *PNLIP*

**Fig. 1:**
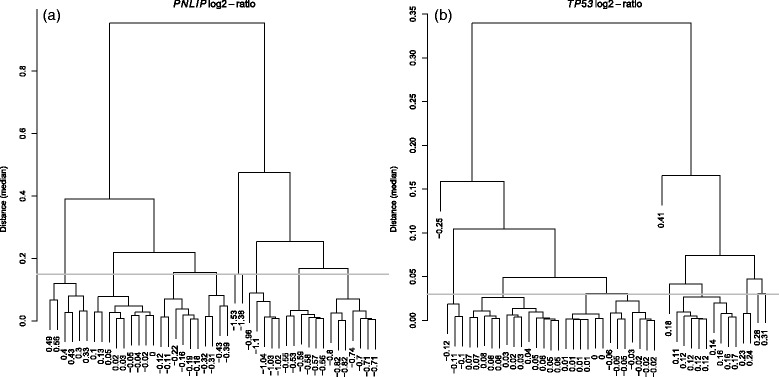
Hierarchical clustering for data division. **a** Tree of paired-ratio values (log2-transformed) of gene *PNLIP*. **b** Tree of paired-ratio values (log2-transformed) of gene *TP53*

**Fig. 2:**
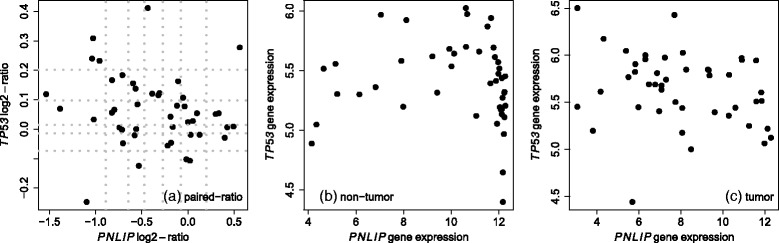
Comparison of expression and paired-ratio between gene *TP53* vs. gene *PNLIP*. **a** Comparison of paired-ratio values (log2-transformed). *Gray dotted lines* represent the cutoff values for defining subsets. **b** Comparison of expression values for non-tumor tissues. **c** Comparison of expression values for tumor tissues

**Fig. 3:**
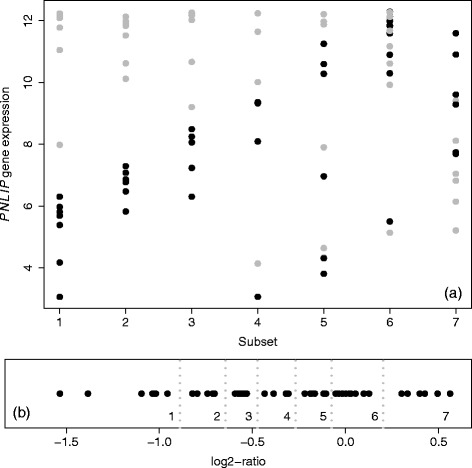
Expression and paired-ratio of gene *PNLIP*. **a** Expression values for tissues in seven subsets (*gray color* represents non-tumor and *dark color* represents tumor). **b** Paired-ratio values (log2-transformed) in seven subsets (*gray dotted vertical lines* for their separation)

The hierarchical clustering tree (with Euclidean distance and the “median” agglomeration method) for the log2-transformed ratio values of gene *PNLIP* is included in Fig. 1 a. Several major clusters of subjects can be generated if we cut the tree at 0.15. After including these isolated subjects into their nearby clusters, we can obtain seven clusters (subgroups of tumor/non-tumor pairs). Therefore, seven subsets of genome-wide expression data were defined accordingly with sample size 7+7, 7+7, 6+6, 4+4, 6+6, 9+9, or 6+6 (see Fig. 2 a). Figure 3 b shows the paired expression ratio values of gene *PNLIP* [log2-transformation applied here for the convenience of visualization of up-regulation (positive sign) or down-regulation (negative sign)]. Figure 3 a shows the individual expression values for gene *PNLIP* in different subsets. Notice that, from Fig. 3 b, subsets 1 represents a clear down-regulation of gene *PNLIP*, and subsets 6 and 7 represents null and up-regulation of gene *PNLIP*, respectively.

### ***z***-Scores based on gene *PNLIP*

**Fig. 4:**
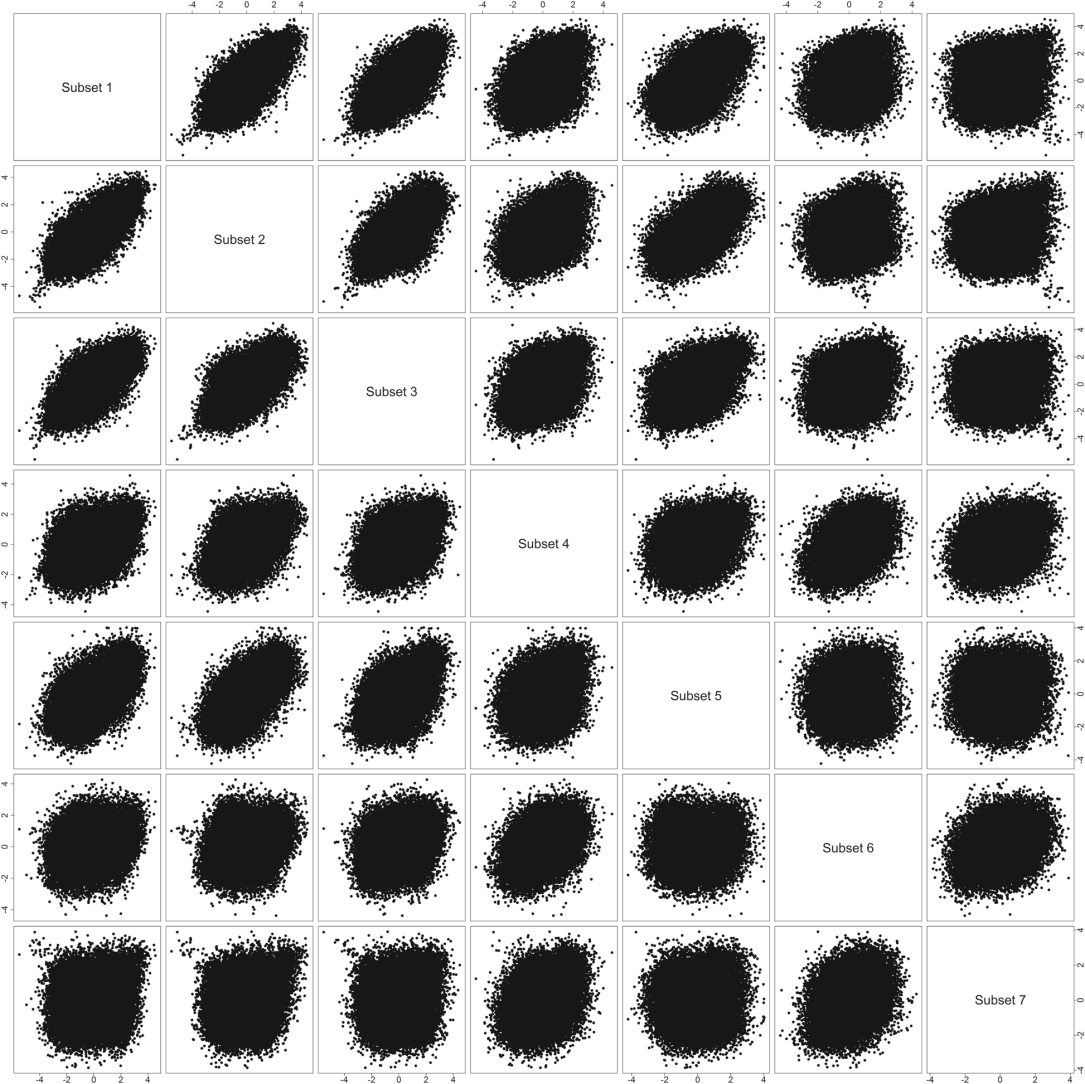
*z*-score comparison (gene *PNLIP*). Pair-wise scatterplots for comparing *z*-scores from seven subsets defined by the paired-ratio of gene *PNLIP*

Figure 4 shows pair-wise scatterplot for comparing *z*-scores from the seven subsets defined by the paired-ratio of gene *PNLIP*. Most scatterplots for adjacent or close-to-adjacent subsets are showing a relatively regular positive correlation pattern (implying overall consistent gene behaviors). The scatterplots for far-from-adjacent subsets are mostly showing an irregular weak correlation pattern (implying a considerable amount of inconsistent gene behaviors). As mentioned above, subsets 1, 6 and 7 are representative for down-regulation, null and up-regulation of gene *PNLIP*, respectively. It is clear that the scatterplot for subsets 7 vs. 1 is showing the most irregular pattern, which implies that many genes have clearly discordant behaviors when gene *PNLIP* changes its behavior from down-regulation to up-regulation.

### Significant pathways based on gene *PNLIP*

**Fig. 5:**
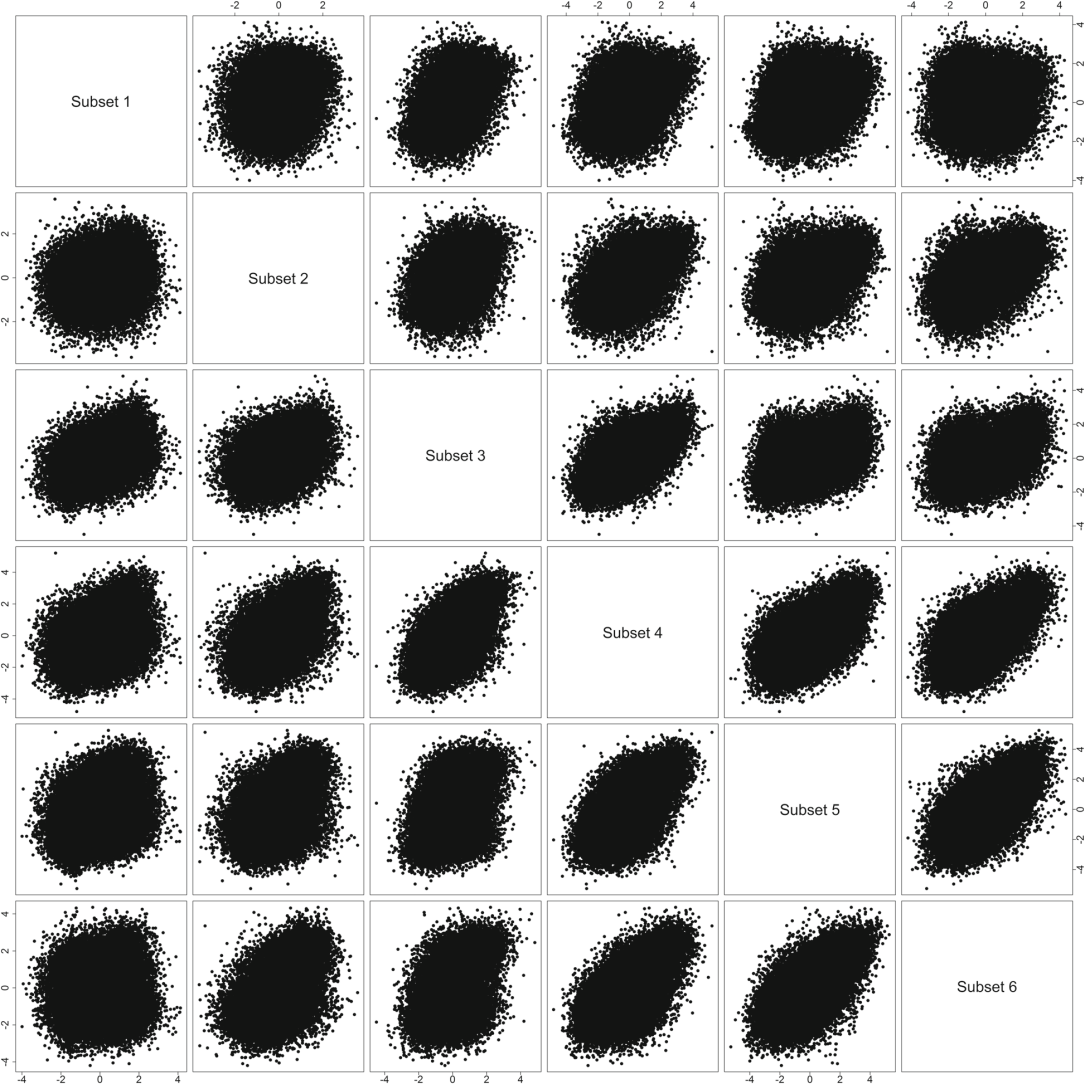
*z*-score comparison (gene *TP53*). Pair-wise scatterplots for comparing *z*-scores from six subsets defined by the paired-ratio of gene *TP53*

Table 2 lists the significant KEGG pathways identified by the discordance enrichment analysis (with *DES*>0.80, also the related maximum *FDR*<0.05). Among these eleven pathways, there are neuroactive ligand receptor interaction, olfactory transduction, alpha-linolenic-acid metabolism and linoleic-acid metabolism pathways. The literature support for the association between pancreatic cancer and each of these pathways will be discussed later. For the olfactory transduction and neuroactive ligand receptor interaction pathways, Fig. 5 shows their *z*-score pattern changes when all the adjacent subsets are pair-wisely compared and three representative subsets (1, 6, 7, see above for their details) are also pair-wisely compared. For the pairs of subsets 2 vs. 1, 3 vs. 2, concordant behaviors can be overall observed for the genes in these two pathways. Discordant behaviors can be overall observed for the pairs 6 vs. 5, 7 vs. 6, 6 vs. 1 and 7 vs. 1. Particularly for the pair 7 vs. 1 (up-regulation vs. down-regulation for gene *PNLIP*), the genes in olfactory transduction pathway are mostly down-regulated in subset 1 but evenly up-regulated or down-regulated in subset 7, and the genes in neuroactive ligand receptor interaction pathway are almost evenly up-regulated or down-regulated in both subsets.

**Table 2:** Pathways identified by the discordance enrichment analysis

KEGG pathway	*DES*	*FDR*
Gene *PNLIP* based analysis		
**Neuroactive ligand receptor interaction**	>0.99	<0.01
**Olfactory transduction**	>0.99	<0.01
Ribosome	>0.99	<0.01
Maturity onset diabetes of the young	>0.99	<0.01
**alpha-Linolenic acid metabolism**	>0.99	<0.01
Glycine serine and threonine metabolism	0.98	<0.01
Steroid hormone biosynthesis	0.97	<0.01
Pentose and glucuronate interconversions	0.96	0.01
Ascorbate and aldarate metabolism	0.95	0.02
**Linoleic acid metabolism**	0.84	0.03
Proximal tubule bicarbonate reclamation	0.81	0.04
Gene *TP53* based analysis		
**Neuroactive ligand receptor interaction**	>0.99	<0.01
**Olfactory transduction**	>0.99	<0.01
Cardiac muscle contraction	0.89	0.04
**alpha-Linolenic acid metabolism**	0.85	0.07
**Linoleic acid metabolism**	0.80	0.09

KEGG pathways with *DES* >0.80 are listed by decreasing order of *DES* with related *FDR*. KEGG pathways in bold font are identified by both analysis (one based on gene *PNLIP* and the other based on gene *TP53*)

### Data division based on gene *TP53*

**Fig. 6:**
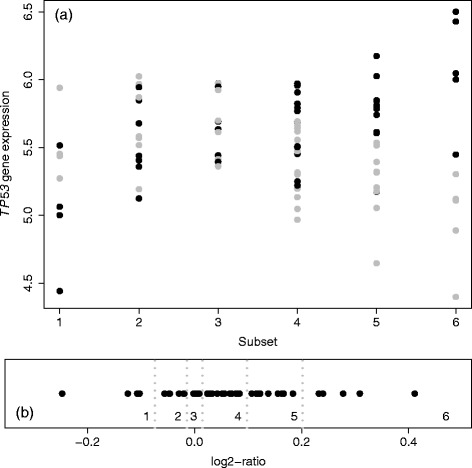
Expression and paired-ratio of gene *TP53*. **a** Expression values for tissues in six subsets (*gray color* represents non-tumor and *dark color* represents tumor). **b** Paired-ratio values (log2-transformed) in six subsets (*gray dotted vertical lines* for their separation)

The hierarchical clustering tree (with Euclidean distance and the “median" agglomeration method) for the log2-transformed ratios values of gene *TP53* is included in Fig. 1 b. Several major clusters of subjects can be generated if we cut the tree at 0.03. After including these isolated subjects into their nearest clusters, we can obtain six clusters (subgroups of tumor/non-tumor pairs). Therefore, six subsets of genome-wide expression data were defined accordingly with sample size 4+4, 7+7, 6+6, 13+13, 10+10, or 5+5 (see Fig. 2 a). Figure 6 b shows the paired expression ratio values of gene *TP53* [log2-transformation applied here for the convenience of visualization of up-regulation (positive sign) or down-regulation (negative sign)]. Figure 6 a shows the individual expression values for gene *TP53* in different subsets. Notice that, from Fig. 6 b, subsets 1 represents a clear down-regulation of gene *TP53*, and subsets 3 and 6 represents null and up-regulation of gene *TP53*, respectively.

### ***z***-Scores based on gene *TP53*

**Fig. 7:**
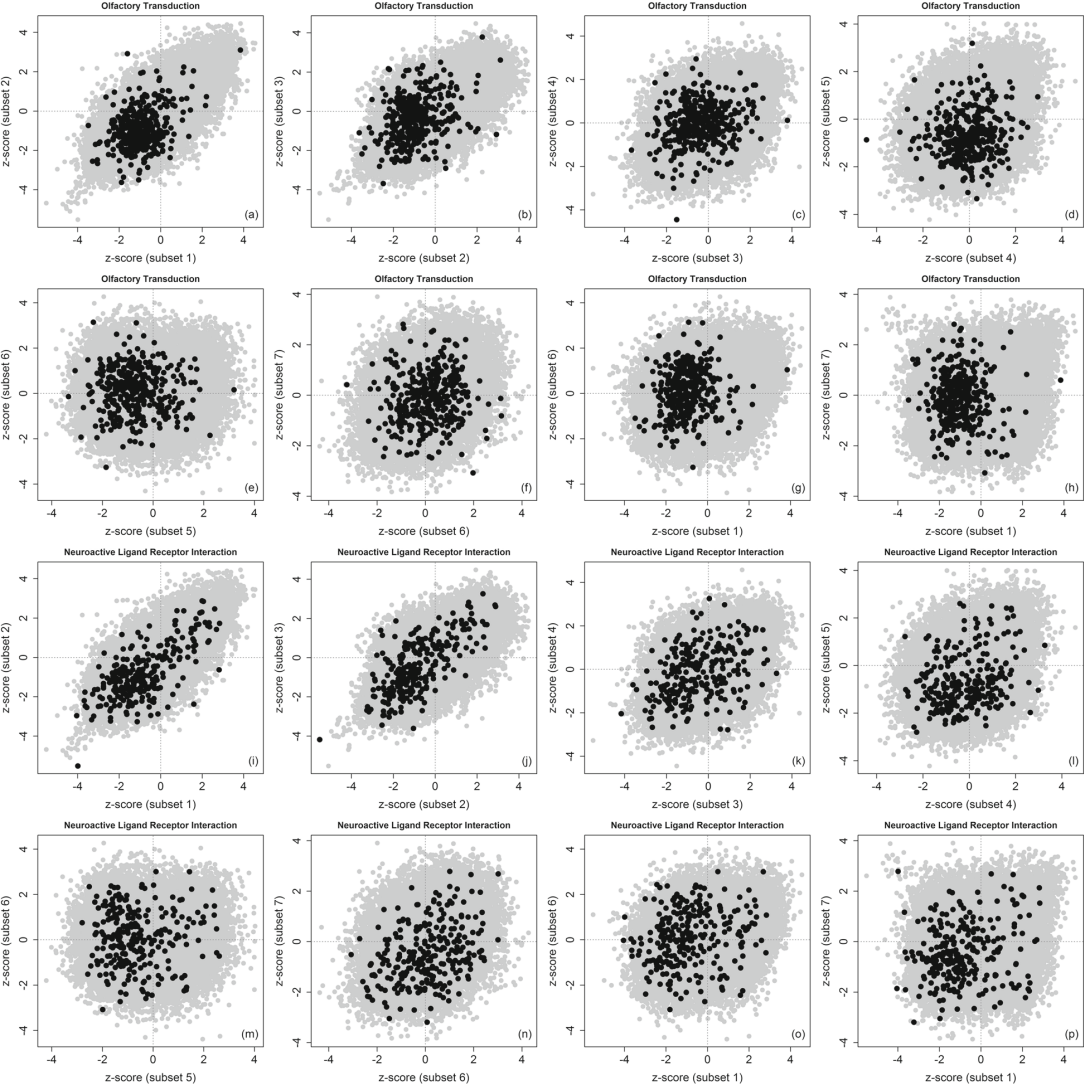
*z*-scores in two most significantly detected pathways (gene *PNLIP*). Pair-wise scatterplots for comparing *z*-scores in the given pathway (*dark color*) and out of the given pathway (*gray color*). All the adjacent subsets are pair-wisely compared (e.g. 2 vs. 1, 3 vs. 2, 4 vs. 3, 5 vs. 4, 6 vs. 5 and 7 vs. 6) and three representative subsets (1 for down-regulation, 6 for null, and 7 for up-regulation) are also pair-wisely compared (7 vs. 6 already shown, then 6 vs. 1 and 7 vs. 1). The order of scatterplots is shown as (**a**-**p**)

Figure 7 shows pair-wise scatterplot for comparing *z*-scores from the six subsets defined by the paired-ratio of gene *TP53*. Many scatterplots for adjacent or close-to-adjacent subsets are still showing a relatively regular positive correlation pattern (implying overall consistent gene behaviors). Almost all the scatterplots for far-from-adjacent subsets are showing an irregular weak correlation pattern (implying a considerable amount of inconsistent gene behaviors). As mentioned above, subsets 1, 3 and 6 are representative for down-regulation, null and up-regulation of gene *TP53*, respectively. All the pair-wise scatterplots for these three subsets are showing irregular patterns (with the scatterplot for subsets 6 vs. 1 the most irregular), which implies that many genes have clearly discordant behaviors when gene *TP53* change its behavior from down-regulation to null, and then to up-regulation.

### Significant pathways based on gene *TP53*

**Fig. 8:**
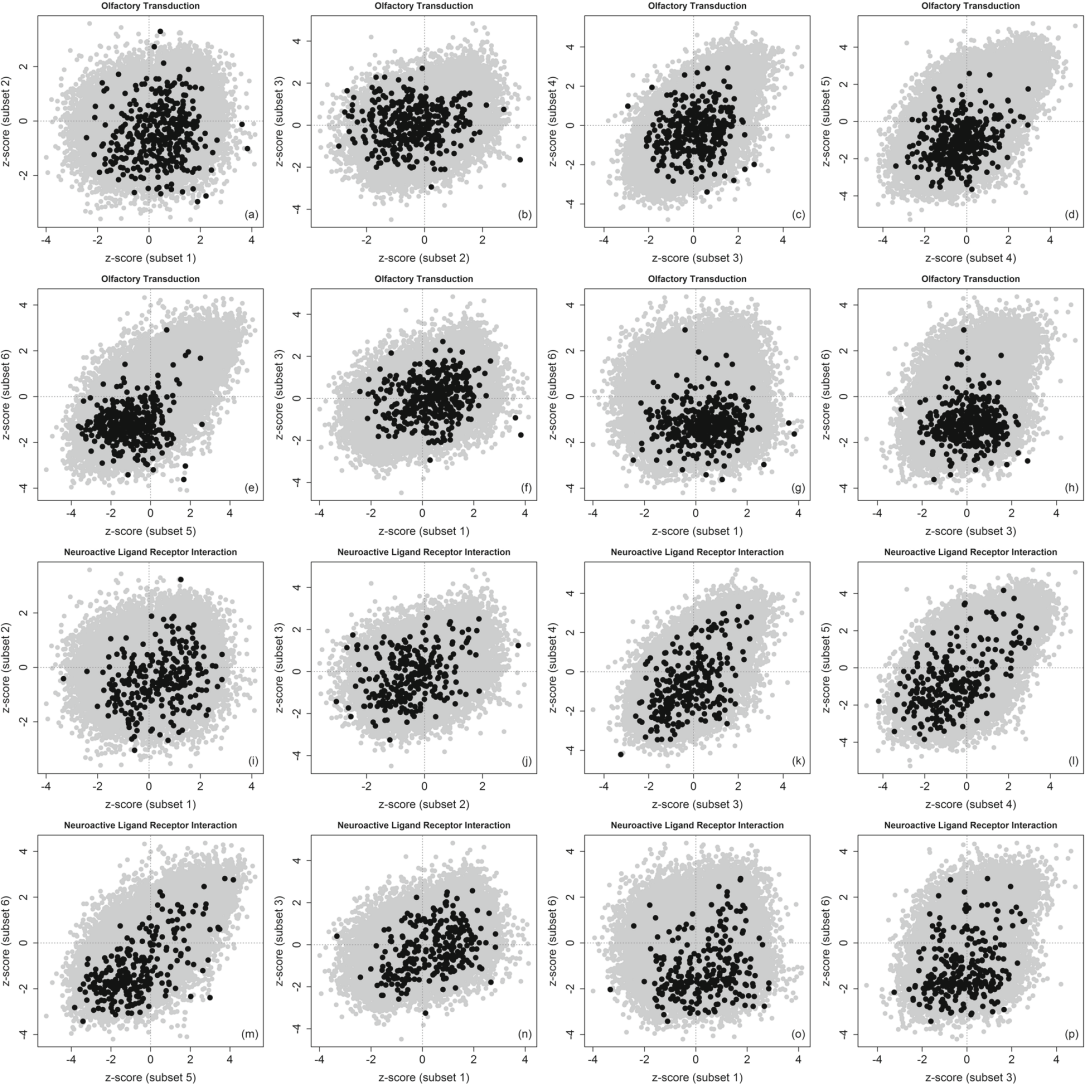
*z*-scores in two most significantly detected pathways (gene *TP53*). Pair-wise scatterplots for comparing *z*-scores in the given pathway (*dark color*) and out of the given pathway (*gray color*). All the adjacent subsets are pair-wisely compared (e.g. 2 vs. 1, 3 vs. 2, 4 vs. 3, 5 vs. 4, and 6 vs. 5) and three representative subsets (1 for down-regulation, 3 for null, and 6 for up-regulation) are also pair-wisely compared (3 vs. 1, 6 vs. 1 and 6 vs. 3). The order of scatterplots is shown as (**a**-**p**)

Table 2 list the significant KEGG pathways identified by the discordance enrichment analysis (with *DES*>0.80, also the related maximum *FDR*<0.10). Among these five pathways, there are neuroactive ligand receptor interaction, olfactory transduction, alpha-linolenic-acid metabolism and linoleic-acid metabolism pathways (which have been identified above by the analysis based on gene *PNLIP*). For the olfactory transduction and neuractive ligand receptor interaction pathways, Fig. 8 shows their *z*-score pattern changes when all the adjacent subsets are pair-wisely compared and three representative subsets (1, 3, 6, see above for their details) are also pair-wisely compared. For the pairs of subsets 6 vs. 5, 5 vs. 4 and 4 vs. 3, concordant behaviors can be overall observed for the genes in these two pathways. Discordant behaviors can be overall observed for the pairs 2 vs. 1, 3 vs. 2, 3 vs. 1, 6 vs. 1 and 6 vs. 3. Particularly for the pair 6 vs. 1 (up-regulation vs. down-regulation for gene *TP53*), the genes in olfactory transduction pathway are mostly down-regulated in subset 6 but evenly up-regulated or down-regulated in subset 1, and the genes in neuractive ligand receptor interaction pathways are somewhat evenly up-regulated or down-regulated in both subsets.

### Literature support

We have conducted a discordance enrichment analysis based on gene *PNLIP* and a discordance enrichment analysis based on gene *TP53*. Among two lists of identified pathways, there are four in common: neuroactive ligand receptor interaction, olfactory transduction, alpha-linolenic-acid metabolism and linoleic-acid metabolism pathways (see Table 2). To further understand these pathways, we have checked the related biomedical literature.

The genome-wide expression data analyzed in this study were collected based on the microarray technology for RNA profiling. Genome-wide association study (GWAS) data have also been collected for pancreatic cancer research based on the microarray technology for DNA profiling (single nucleotide polymorphism, or SNP). Wei et al. [32] recently conducted a pathway analysis for a large GWAS data on pancreatic cancer research. They reported only two pathways. Interestingly, these two pathways are neuroactive ligand receptor interaction and olfactory transduction pathways (top two identified from both of our analysis results, see above for details). Notice that their findings were based on a different type of molecular data. This is a strong support for the discordance enrichment analysis results.

We also found at least one support for both alpha-linolenic-acid metabolism and linoleic-acid metabolism pathways. Wenger et al. [33] conducted a study on the roles of alpha-linolenic acid (ALA) and linoleic acid (LA) on pancreatic cancer and they observed an association between the disease and these two fatty acids.

### Insignificant pathways

**Fig. 9:**
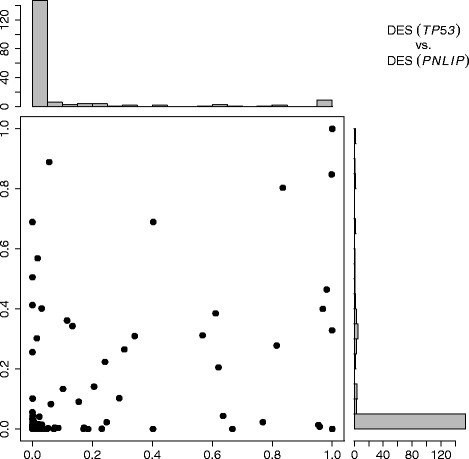
Comparison of *DES* between gene *TP53* vs. gene *PNLIP*. (*left, lower*) Scatterplot of *DES* based on gene *TP53* vs. *DES* based on gene *PNLIP*, notice that there are overlapped dots in the scatterplot. (*left, upper*) Histogram of *DES* based on gene *TP53*. (*right, lower*) Histogram of *DES* based on gene *PNLIP*

Figure 9 shows 186 *DES* based on *PNLIP* vs. *DES* based on *TP53*. These two lists of *DES*’s are highly correlated (Spearman’s rand correlation 0.642), although some pathways identified in the analysis results based on *PNLIP* are not significant in the analysis results based on *TP53*. (Notice that a pathway with *DES*∼1 is significantly enriched in clearly discordant behaviors; and a pathway with *DES*∼0 is evidently not enriched in clearly discordant behaviors.) Only a small number of pathways were identified by the discordance enrichment analysis. The histograms in the figure show that most pathways are showing insignificant *DES*’s. For each of two analysis results, there are more than 140 pathways (among 186) with *DES*<0.05. The number of pathways with both *DES*<0.01 or both *DES*<0.05 is 111 (60%) or 138 (74%), respectively. For both *DES*<0.25, 0.5 or 0.75, there are 154 (83%), 164 (88%) or 173 (93%) pathways, respectively. Therefore, most pathways are evidently not enriched in clearly discordant behaviors among the series of subsets defined by the paired expression ratio of gene *PNLIP*; neither are they among the series of subsets defined by the paired expression ratio of gene *TP53*. Many disease related pathways have been listed by KEGG (http://www.genome.jp/kegg/pathway.html). The collection of pancreatic cancer related pathways (or KEGG pancreatic cancer) and the collection of cancer related pathways (or KEGG pathways in cancer) are not enriched from both analysis results (*DES*<0.001). Among the pathway components of these two collections (e.g. cell cycle pathway, apoptosis pathway, etc.), the highest *DES* value is <0.01 for the PPAR signaling pathway from the analysis results based on *PNLIP*, and the highest *DES* value is <0.05 for the cytokine-cytokine receptor interaction pathway from the analysis results based on *TP53*. Pathways like hedgehog signaling, proteasome, and primary immunodeficiency are also showing low *DES* values (all <0.05).

### Expression profiles of *PNLIP* vs. *TP53*

*PNLIP* is a gene shown recently its association with pancreatic cancer [22]. *TP53* is a well-known tumor suppressor gene. From the above comparison, it is interesting that the discordance enrichment analysis results based on *PNLIP* are highly correlated with the discordance enrichment analysis results based on *TP53*. To further understand this correlation, we compared the expression profile of *PNLIP* with the expression profile of *TP53*. Figure 2 a shows a relatively weak negative correlation (Spearman’s rank correlation -0.250) between two lists of paired-ratios but the correlation is not statistically significant (*p*-value=0.098). In the non-tumor group (Fig. 2 b), the negative correlation (Spearman’s rank correlation -0.318) achieves a *p*-vlaue 0.033. In the tumor group (Fig. 2 c), the negative correlation (Spearman’s rank correlation -0.276) is again not statistically significant (*p*-value=0.066). Furthermore, the ratio cutoff values for defining subsets were added to Fig. 2 a. A contingency table can be generated according to these grids (for example, the cell number is one for row one and column one in the table). The chi-square test for this sparse contingency table is not statistically significant (simulation based *p*-value >0.3). Therefore, in summary, gene *PNLIP* may be negatively associated with gene *TP53* but no clear statistical significance has been observed in this study.

### Comparison to gene set analysis

Efron and Tibshirani [34] have proposed a gene set analysis (GSA) method for analyzing enrichment in pathways (or gene sets). It was suggested by Maciejewski [35] that this method is preferred in a gene set enrichment analysis. In some situations of integrative data analysis, different data sets cannot be simply pooled together. For each data set, the *p*-value of enrichment in up-regulation can be obtained for each gene set. To integrate the *p*-values from multiple data sets (for the same gene set), we can consider Fisher’s method (Fisher’s combined probability test). log-Transformed *p*-values are summed up and then multiplied by -2, which is well-known to follow a chi-squared distribution under the null hypotheses. In this way, we can perform an integrative gene set enrichment analysis of multiple data sets (when different data sets cannot be pooled together). Gene sets (or pathways) can be ranked by their chi-squared *p*-values. (Similarly, the *p*-value of enrichment in down-regulation can also be obtained by GSA for each gene set and each data set. Then, the related chi-squared *p*-values can be calculated by Fisher’s method.) Notice that, our analysis purpose is to detect discordance enrichment among multiple data sets. However, the discordance feature is usually not considered in a traditional integrative analysis.

In this study, our analysis results were based on several subsets divided from a genome-wide expression data set with a relatively large sample size. These subsets could be pooled back (to be the original large data set). Therefore, we applied GSA to the original data (so that we could take the advantage of its relatively large sample size). However, after considering the adjustment for multiple hypothesis testing, no pathways (or gene sets) could be identified even at the false discovery rate 0.3 (or *FDR*<30*%*). (Therefore, the detail of GSA results is not reported).

## An application to The Cancer Genome Atlas (TCGA) data sets

For a further illustration of our method, we performed a discordance enrichment analysis of the RNA sequencing (RNA-seq) data collected by The Cancer Genome Atlas (TCGA) project [3]. At the time of study, with the consideration of adequate numbers of normal/tumor subjects, we selected the RNA-seq data for studying prostate adenocarcinoma (PRAD), colon adenocarcinoma (COAD), stomach adenocarcinoma (STAD), head and neck squamous cell carcinoma (HNSC), thyroid carcinoma (THCA) and liver hepatocellular carcinoma (LIHC). Among these different types of diseases, we expected a certain level of dissimilarity in genome-wide expression profiles. Therefore, we applied our method to these six TCGA RNA-seq data sets (and our proposed two-level mixture model was useful to reduce the number of model parameters). Gene expression profiles for more than 20,000 common genes were available for our analysis.

Among 186 KEGG pathways, we report the analysis results for a collection of cancer related pathways. There are sixteen of these pathways in KEGG but fourteen of them are available in the Molecular Signatures Database [7, 8]. In Table 3, the discordance enrichment analysis results are also compared to the results based on GSA-based Fisher’s method (see *Comparison to Gene Set Analysis* for details). However, it is important to emphasize that the detection of discordance enrichment is our focus in this study and the feature of discordance is usually not considered in a traditional integrative analysis (e.g. Fisher’s method).

**Table 3:** A comparison study

KEGG pathway	*p*-value (GSA-Fisher, up)	*p*-value (GSA-Fisher, down)	*DES*
ECM RECEPTOR INTERACTION	0.099	0.843	0.256
CYTOKINE CYTOKINE RECEPTOR INTERACTION	0.774	0.708	0.954
FOCAL ADHESION	0.239	0.432	>0.999
WNT SIGNALING PATHWAY	0.873	0.119	0.995
ADHERENS JUNCTION	0.674	0.454	0.999
JAK STAT SIGNALING PATHWAY	0.696	0.701	0.723
MAPK SIGNALING PATHWAY	0.980	0.130	>0.999
MTOR SIGNALING PATHWAY	0.434	0.475	0.999
PPAR SIGNALING PATHWAY	0.997	<0.001	>0.999
VEGF SIGNALING PATHWAY	0.293	0.868	>0.999
APOPTOSIS	0.399	0.388	>0.999
P53 SIGNALING PATHWAY	<0.001	>0.999	0.104
CELL CYCLE	<0.001	>0.999	<0.001
TGF BETA SIGNALING PATHWAY	0.970	0.090	0.976

For significant detections, check pathways with low *p*-values or pathways with high *DES*

**Fig. 10:**
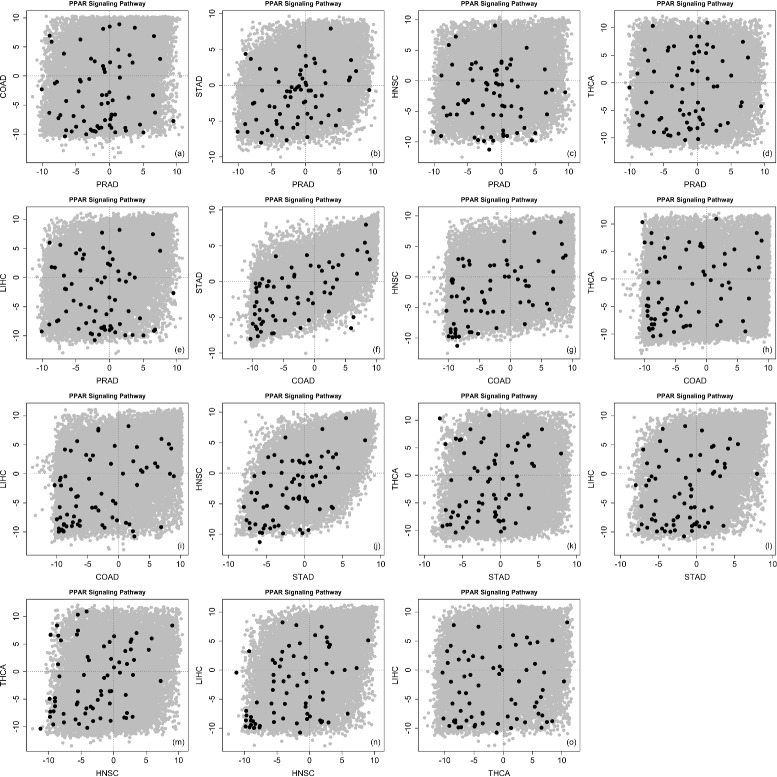
*z*-scores in PPAR signaling pathway (TCGA data). Pair-wise scatterplots for comparing *z*-scores in the given pathway (*dark color*) and out of the given pathway (*gray color*). *x*-Axis and *y*-axis represent *z*-scores for different types of diseases. The order of scatterplots is shown as (**a**-**o**)

**Fig. 11:**
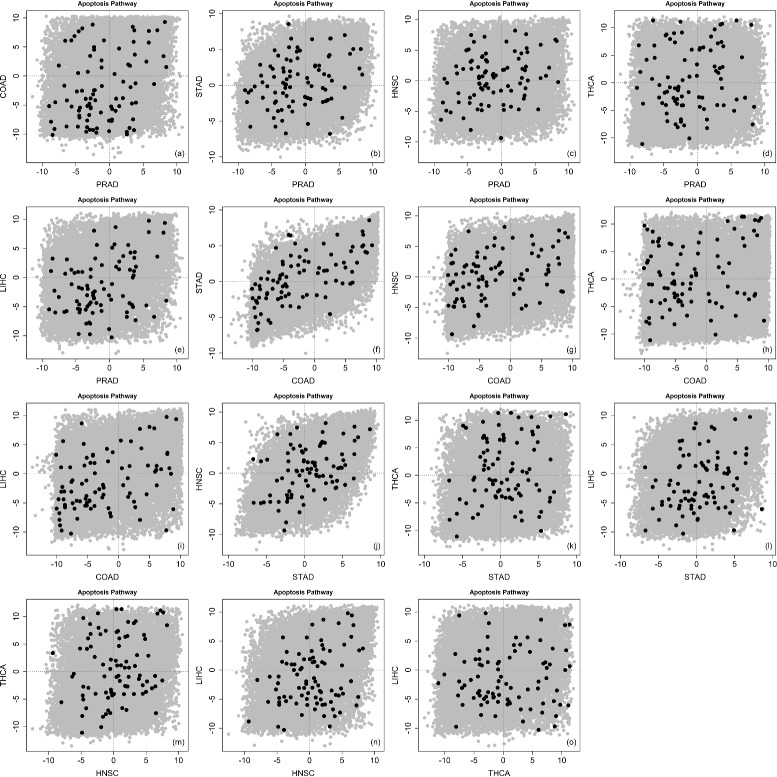
*z*-scores in apoptosis pathway (TCGA data). Pair-wise scatterplots for comparing *z*-scores in the given pathway (*dark color*) and out of the given pathway (*gray color*). *x*-Axis and *y*-axis represent *z*-scores for different types of diseases. The order of scatterplots is shown as (**a**-**o**)

Table 3 shows the comparison of our discordance enrichment scores (*DES*) to the *p*-values calculated by GSA-based Fisher’s method (up-regulation or down-regulation). (Lower *p*-value for more significant result but higher *DES* for more significant result.) The p53 signaling pathway, cell cycle pathway, and PPAR signaling pathway are three pathways with significant GSA-Fisher *p*-values. For the p53 signaling pathway and cell cycle pathway, their *DES* suggest low discordance among different types of diseases for these two well-known pathways. For the PPAR signaling pathway, its *DES* is also highly significant. Figure 10 shows a considerable amount of concordance as well as a considerable amount of discordance among different types of diseases for this pathway. With the consideration of either Bonferroni-type adjustment or FDR-type adjustment, no detection can be further observed based on GSA-based Fisher’s method. However, our method identified a few pathways with significant discordance enrichment (*DES*>0.999) including the focal adhesion, MAPK signaling, VEGF signaling and apoptosis pathways. Figure 11 shows a considerable amount of discordance among different types of diseases for the well-known apoptosis pathway. Furthermore, the WNT signaling, adherens junction, MTOR signaling and TGF-beta signaling pathways are also showing high *DES*, which suggest possible discordance enrichments for these pathways.

## Conclusions

In this study, we suggested a discordance gene set enrichment analysis for a series of two-sample genome-wide expression data sets. To reduce the parameter space, we proposed a two-level multivariate normal distribution mixture model. Our model is statistically efficient with linearly increased parameter space when the number of data sets is increased. Then, gene sets can be detected by the model-based probability of discordance enrichment.

Based on our two-level model, if the proportion of complete concordance component is high, then more genes behave concordantly among different data sets. Similarly, if the proportion of complete independence component is high, then more genes behave discordantly among different data sets. In the complete concordance component (model), only complete concordant behaviors are considered: all “up," all “down" or all “null." Therefore, there are only three items *j*=0,1,2 for the outer summation term. For each complete concordant behavior, we have independence among different data sets. Statistically, conditional on a underlying complete concordant behavior (with probability *π*_*j*_), we have an inner product term of probability density functions calculated based on different data sets. In the complete independence component (model), genes behave completely independent among different data sets, which is reflected in the outer product term. For each data set, the underlying behavior for each gene can be “up," “down" or “null." However, the behavior cannot be directly observed and the related probability density function is calculated based on a mixture model.

Our method was applied to a microarray expression data set collected for pancreatic cancer research. The data were collected for forty-five matched tumor/non-tumor pairs of tissues. These pairs were first divided into seven subgroups for defining seven subsets of genome-wide expression data, according to the paired expression ratio of gene *PNLIP*. This gene was recently shown its association with pancreatic cancer. Our purpose was to understand discordance gene set enrichment when gene *PNLIP* changes its behavior from down-regulation to up-regulation. Among a few identified pathways, the neuroactive ligand receptor interaction, olfactory transduction pathways were the most significant two. The alpha-linolenic-acid metabolism and linoleic-acid metabolism pathways were also among the list. To better understand these results, we divided again the original data with forty-five pairs of tumor/non-tumor tissues into six subsets, according to the paired expression ratio of gene *TP53* (a well-known tumor suppressor gene). The above four pathways were also identified by the discordance gene set enrichment analysis, with the neuroactive ligand receptor interaction, olfactory transduction pathways still the most significant two. After our literature search, we found that these two pathways were the only two identified for their association with pancreatic cancer in a recent independent pathway analysis of genome-wide association study (GWAS) data. For the alpha-linolenic-acid metabolism and linoleic-acid metabolism pathways, we found a previous study that the association between pancreatic cancer and these two fatty acids (alpha-linolenic acid and linoleic acid) was observed.

A few discordant behaviors from individual genes can be observed from Figs. 7 and 8. In Fig. 7 p, among genes in the neuroactive ligand receptor interaction pathway (black dots), a gene with the most negative *z*-score in subset 1 has the most positive *z*-score in subset 7. This is a clear change from down-regulation to up-regulation. In Fig. 8 a-b, among genes in the olfactory transduction pathway (black dots), a gene with the most positive *z*-score in subset 2 has a moderately positive *z*-score in subset 1, but its *z*-score in subset 3 is clearly negative. This is a clear change from up-regulation to down-regulation.

We conducted a discordance gene set enrichment analysis based on gene *PNLIP* and a discordance gene set enrichment analysis based on gene *TP53*. Only a few among 186 KEGG pathways were identified. Most pathways (like cancer and pancreatic cancer related pathways) were evidently not enriched in discordant gene behaviors. This suggest unique molecular roles of both genes *PNLIP* and *TP53* in pancreatic cancer development. There were four pathways identified from both analysis results and we found biomedical literature to support the association between pancreatic cancer and these pathways. Some pathways identified in one analysis were not identified in the other analysis. It is also biologically interesting to understand these pathways.

It was biologically interesting to observe pathways with clearly discordant gene behaviors when the paired expression ratio of an important disease-related gene was changing. The analysis results in this study illustrated the usefulness of our proposed statistical method. Our method was developed based on *z*-scores that are statistical measures of differential expression, and many existing two-sample statistical tests could be used for generating *z*-scores. Therefore, in this study, we demonstrated our method based on a partition of a relatively large two-sample microarray data set as well as several two-sample genome-wide expression data sets collected by the recent RNA-seq technology.

Our method is statistically novel for its two-level structure, which is developed based on a biological motivation (genes’ behaviors among different data sets). Due to this two-level structure, the parameter space of our model is increased linearly when the number of data sets is increased. Then, the parameter estimates can be statistically efficient. In our mixture model, conditional independence is the key to reduce the complexity of multivariate data analysis. For each gene, when the mixture component information is given for all the data sets, its *z*-scores are independent. (Notice that there is no overlap among multiple data sets). Mathematical and computational convenience is achieved for our statistical model due to this unique feature.

Our method is based on the well-established mixture model framework and the Expectation-Maximization (EM) algorithm for parameter estimation. One limitation is that the proposed three-component mixture model may not fit *z*-scores well for some data. This can be improved by considering more components in the mixture model. For example, instead of a simple consideration of down-regulation, null and up-regulation, we may consider more components like strong-down-regulation, weak-down-regulation, null, weak-up-regulation and strong-up-regulation. This will only proportionally increase the parameter space (still linear with the number of data sets for our two-level mixture model).

It is also interesting to extend our method for more complicated analysis purpose. For example, we may be interested in identifying trend changes (monotonically increasing or decreasing) instead of general changes. Also, for example, we may have multiple data sets collected for different disease stages, but the data set for normal/reference/control stage is not large enough to be divided and it has to be used repeatedly in two-sample comparisons (then *z*-scores are not even conditionally independent). For these situations, the extension of our method would require a considerable amount of research effort.
